# Powered Toothbrushes: An Opportunity for Biofilm and Gingival Inflammation Control

**DOI:** 10.1155/2022/6874144

**Published:** 2022-08-22

**Authors:** Cassiano Kuchenbecker Rösing, Eduardo Garduño, Sandra Kalil Bussadori, Agustín Zerón, Paulo Vinícius Soares, Marc Saadia, Cristina Cunha Villar

**Affiliations:** ^1^Department of Periodontology, Faculty of Dentistry, Federal University of Rio Grande do Sul, Porto Alegre, Brazil; ^2^Orthodontic Private Practice, Mexico City, Mexico; ^3^Post-Graduate Program in Rehabilitation Sciences, UNINOVE – Universidade Nove de Julho, São Paulo, Brazil; ^4^Editor Journal of the Mexican Dental Association, Mexico City, Mexico; ^5^Department of Operative Dentistry and Dental Materials, Federal University of Uberlandia, Uberlândia, Brazil; ^6^Private Practice, Mexico City, Mexico; ^7^Division of Periodontology, University of São Paulo, São Paulo, Brazil

## Abstract

The present review aimed at a broad investigation on the potential of powered as compared to manual toothbrushes in different aspects of clinical dentistry. Studies evaluating plaque and gingival inflammatory parameters were included, as well as those that investigated adverse effects. Emphasis was given separately to adults, youngsters, special-needs patients, and those under fixed orthodontic therapy. In general, comparisons favored powered toothbrushes. In summary, approximately 68% of the included studies, in terms of plaque/gingival inflammation in adults, presented better results for powered toothbrushes. In children and special-needs populations, approximately 40% of the included studies favored powered toothbrushes for plaque/gingival inflammation, and none favored manual ones. In orthodontic individuals, 50% of the studies also demonstrated a better effect of powered toothbrushes on plaque and gingival inflammation. All included studies that assessed adverse events did not demonstrate a difference in these effects when comparing manual vs. powered toothbrushes. It is concluded that the use of powered toothbrushes is an opportunity to enhance patterns of plaque control and associated gingival inflammation.

## 1. Introduction

Supragingival plaque control is one of the most beneficial preventive strategies in clinical dentistry. In accordance with individual needs, the dental practitioner is responsible for motivating and instructing the patient to carry out more effective plaque control, always minimizing adverse effects. Studies consistently demonstrate that individuals with good standards of oral hygiene standards and good professional maintenance of oral health have a lower incidence of dental caries, periodontal diseases, tooth loss, and as shown more recently, better outcomes with dental implants.

A classic study performed in Sweden by Axelsson et al. clearly demonstrated that a strict oral hygiene program results in long-term maintenance of the teeth with a lower incidence of caries and periodontal breakdown [1]. Other studies also have demonstrated similar findings [2, 3]. Despite the well-established positive role of proper plaque control, optimal levels of oral hygiene are not observed in the population as a whole [4, 5].

Manual toothbrushes are the instruments most widely used for the removal of plaque. Although initially developed for situations where manual toothbrushes are not effective, especially due to impairment of motor function, powered toothbrushes are currently used as an alternative to manual toothbrushes.^1^ The industry has developed different types of powered toothbrushes, and this innovative technology has evolved very rapidly. In 1998, the European Workshop on Mechanical Plaque Control carefully reviewed the evidence supporting the use of powered toothbrushes. Its conclusions included the increased ability of powered toothbrushes to remove plaque from the interproximal area; the importance of professional motivation (reinforcement) to achieve optimal results with powered toothbrushes; the safety of powered toothbrushes regarding lack of adverse effects in terms of trauma; and superior efficacy of powered toothbrushes as compared to manual ones, as demonstrated in clinical trials. The panel also concluded that toothbrush design could have a role in these results [6].

The literature supporting the use of powered toothbrushes has continuously grown. Continuous updates are needed to keep up with research and development in this area. In the present study, a panel of experts from Brazil and Mexico sought to review the best available evidence on possible indications for powered toothbrushes in clinical dentistry as of December 2020.

This is not a systematic review, since there are multiple questions and interests. Rather, it is a narrative summary of the available, published evidence comparing manual and powered toothbrushes. The authors are aware that publication bias is present and that much of this body of evidence is industry-funded. It is also acknowledged that further research and development in healthcare technologies are of utmost importance.

To compile this evidence, a systematic search of the PubMed database, combining terms of interest for the present study to find all possible articles comparing manual and powered toothbrushes, was conducted. Different terms of interest were combined in each of the topics (always having the outcome and synonyms and the exposure—manual and electric toothbrushes and synonyms). The authors scrutinized the retrieved potential articles for eligibility. For the purposes of this review, *powered toothbrushes* are defined as all toothbrushes that are driven by an external power source. Due to the broad spectrum of this review, the authors gave preference to clinical trials. However, in order to adopt a mechanistic approach, in vitro studies that were considered to shed some light on the theme were also included. It should also be noted that as clinical trials are included in the present review, it is of utmost importance to include information related to adverse events. This was the case in the present review, which includes possible adverse effects, especially the development of noncarious cervical lesions.

### 1.1. Effect of Powered Toothbrushes on Dental Biofilm/Microbial Parameters

One of the main objectives of toothbrushing is to control dental plaque buildup and, consequently, gingival inflammation. In this respect, systematic reviews with meta-analyses are highlighted to investigate the effects of manual versus powered toothbrushes on dental plaque and gingival inflammatory signs. In the study by Jager et al. [7], the results of a meta-analysis of 1,780 subjects indicated that high-frequency, high-amplitude, sonic-powered toothbrushes decreased plaque and gingivitis with significantly better effectiveness than manual toothbrushes.

Different studies have been performed to assess the effect of powered toothbrushes on microbial biofilms, using different laboratory techniques. Several technologies have been incorporated into powered toothbrushes, and a significant effect has been demonstrated for some.  Table 1, for example, captures the effect of a sonic technology for powered toothbrushes against oral bacteria. It is important to understand the effects of such toothbrushes in the context of the mechanisms against oral biofilms.

### 1.2. Effect of Powered Toothbrushes on Dental Plaque and Associated Gingival Inflammatory Parameters in Adults

The use of powered toothbrushes has mainly been studied in adult individuals. To obtain high-quality evidence, randomized controlled clinical trials have been performed. A systematic search of the literature, including keywords related to gingivitis, gingival inflammation, and powered/powered toothbrushes, yielded 22 studies that met eligibility criteria for inclusion in this review (Table 2). From these studies, the industry has financed a significant part; however, approximately half of them are independent studies, as indicated in the conflict of interests in the publications.

A synthesis of qualitative information from these studies, chosen by presenting the comparison of interest, is presented in Table 2. Of 22 randomized clinical trials comparing different powered to manual toothbrushes, 15 (68%) demonstrated the superiority of powered toothbrushes to reduce gingival inflammation. The majority of these trials also analyzed plaque buildup and a similar tendency was observed, in which powered toothbrushes had a better effect. Only two studies (9%) demonstrated a better effect of the manual toothbrush on signs of gingival inflammation. The remaining 5 studies did not reveal statistically significant differences between powered and manual toothbrushes on gingival inflammatory parameters. When parameters related to dental plaque are observed, the same tendency of better results for powered toothbrushes is demonstrated. It should be noted that some studies have also assessed probing depth (PD), which is considered a sign of gingival inflammation as well. In this sense, since alterations in PD are possible with an increase/decrease in inflammation, this was considered in the present review.

It needs to be stressed that the present review aims to provide a general overview of the different effects of powered toothbrushes as compared to manual ones. Therefore, the inclusion of different types of brushes with different designs, several methods of analysis of both plaque and gingival inflammation, and observation periods prevent the pooling of results for meta-analysis. Summarizing the results, it could be stated that powered toothbrushes consistently provide better plaque removal and a reduction of associated gingival inflammation as compared to manual toothbrushes in studies performed on healthy adult individuals.

### 1.3. Adverse Effects of Powered versus Manual Toothbrushes: Possible Relationship between Toothbrush Type and Noncarious Lesions

Toothbrushing has increased worldwide as a result of oral hygiene awareness programs. One of the problems arising from this change in behavior is the occurrence of adverse effects, especially gingival recession and noncarious lesions. Studies assessing the potential impact of powered versus manual toothbrushing on the incidence of gingival recession are lacking.

The diagnosis of noncarious lesions is part of routine dental practice. These multifactorial conditions are usually related to the patient's lifestyle, including the type of toothbrush they use. Few studies have sought to evaluate the relationship between different toothbrush types/brushing habits and the development of gingival recession, tooth abrasion, dentin hypersensitivity, and other noncarious lesions.

Although it is important to study the type of toothbrush used, it is also essential to note that differences in the results obtained with different toothbrushes are also modified by brushing variables, such as timing, frequency, and brushing force. In addition, variations in bristle characteristics, such as filament stiffness and end-rounding, have been assumed to influence factors such as hard and soft tissue abrasion [34].

Even though anecdotal information is available from studies related to toothbrushing and adverse events, the body of evidence is still weak, with the majority of studies not evaluating these parameters. Some systematic reviews suggested that there were insufficient data to support or refute the association between different toothbrushes and noninflammatory gingival recession [35]. Most studies claim that the use of powered toothbrushes implies that less force is applied while brushing. However, very few of these studies have shown a significant difference in the incidence or regression of noncarious lesions such as gingival recession, even with less force applied while brushing. This information is also available from a study that solely used powered toothbrushes. It should be emphasized that a very low number of studies have assessed adverse events related to powered toothbrushes. Therefore, the findings from this study are considered warranted. [36].

When the act of toothbrushing is assessed in isolation (excepting cases of abusive use, e.g., excess use of abrasive dentifrice), clinically significant effects on tooth surface loss are seen [37]. Gingival abrasions can be found in clinical trials of both manual and powered toothbrushes [38].

Therefore, additional laboratory and clinical trials are needed to better evaluate the role of toothbrush type in the development of noncarious lesions (35). What is already known is that individual use of inappropriate brushing techniques is more closely related to the onset of noncarious lesions than the type of toothbrush itself [38].

Five studies included in the present review assessed this potential role. A summary is given in Table 3.

### 1.4. Effect of Powered Toothbrushes on Dental Plaque in the Youngster Population

Our search of the available literature (Table 4) yielded 5 studies that comparatively evaluated powered versus manual toothbrushes in youngsters. As a whole, there is moderate-quality evidence that powered toothbrushes provide a greater benefit than or benefit equal to manual toothbrushes for children. Both powered and manual toothbrushes remove plaque with reasonable efficacy in this population. Regarding plaque control, 2 of the 5 included studies demonstrated the superiority of powered toothbrushes over manual ones in reducing plaque in children. Of the remaining 3 studies, all described powered and manual toothbrushes as equivalent in this respect. However, within the analyses of individual studies, powered toothbrushes generally tended to perform better. However, the clinical significance of these results remains unclear, and additional research is warranted. It is worth mentioning that this result refers to data collected from various types and brands of powered toothbrushes, including toothbrushes with oscillating, rotating, sonic, and ionic actions; that the age of the patients ranged between 6 to 25 years; and that brushing techniques and brushing time varied.

### 1.5. Effect of Powered Toothbrushes on Dental Plaque in Special-Needs Populations

Optimal levels of plaque control are of utmost importance at all ages to achieve adequate levels of oral health. However, mechanical strategies for plaque control often fail to achieve the desired or optimal levels. This is especially true for individuals that lack motor skills or that have special needs. For these reasons, powered toothbrushes have a definite indication for patients lacking fine motor skills, such as those with certain disabilities and autism [47].

The adequacy of plaque control depends on patient adherence. In physically or mentally disabled individuals, difficulties in oral hygiene maintenance are common enough that oral hygiene practices may need to be simplified or modified to suit the individual situation [48].

In the present review, it is inferred that there is moderate-quality evidence that powered toothbrushes provide a greater or equal benefit than manual brushes in special-needs patients. Indeed, both manual and powered toothbrushes decrease levels of plaque and gingival inflammation in this population. Among the 7 clinical trials comparing the effects of powered and manual toothbrushes on plaque control in special-needs individuals, which were included in this review (Table 5), 3 studies demonstrated a superiority of powered over manual toothbrushes in reducing plaque and gingival inflammation. In the remaining 4 studies, an equivalence between powered and manual toothbrushes (or individualized manuals) was observed. It is noteworthy that this result refers to data collected from various types of powered toothbrushes, including toothbrushes with oscillating, rotating, sonic, and ionic actions, and that the age of the patients ranged widely (6 to 34.5 years), as did brushing techniques and times. It must however be taken into account that the type and level of disability of the participants of the included studies also varied. In this sense, each work should be carefully read and understood, with the clinical perspective of to whom these results should apply.

The results encountered herein demonstrate a tendency toward better results with the use of powered toothbrushes by individuals with special needs. This result should be interpreted in light of the concept of personalized oral care, in which each individual needs to be evaluated and monitored over time in order to understand the clinical benefit of each preventive or therapeutic measure.

### 1.6. Effect of Powered Toothbrushes on Dental Plaque/Gingival Inflammatory Signs or Demineralization in Orthodontic Patients

Fixed orthodontic appliances unquestionably increase the difficulty of self-performed mechanical plaque control. In this sense, all attempts to enhance the quality of oral hygiene should be pursued, including the use of powered toothbrushes. This review included 8 studies assessing this population (Table 6). The vast majority of studies (all but one) evaluated the effect on plaque control and/or gingival inflammatory signs. The remaining studies [54] focused on dental caries and demineralization of enamel surfaces. Half of the studies (including the one focusing on demineralization) demonstrated the superiority of powered toothbrushes compared to manual toothbrushes. Three studies demonstrated the equivalence of the effect of powered and manual toothbrushes [58–60], and one study [55] showed a better effect of manual toothbrushes.

Similar to the other topics of the present review, even though there is no consensus, there is a trend toward better effects with powered toothbrushes. These results should always be interpreted with caution in terms of effect size. In this topic, motivation has also been studied, and it has been suggested that powered toothbrushes present an interesting result in this respect, suggesting superiority. In summary, there is a role for powered toothbrushes for individuals under fixed orthodontic therapy.

## 2. Concluding Remarks

The present narrative review of the literature sought to increase awareness among dental care practitioners about the effects of powered toothbrushes as compared to manual ones. It is well known that oral hygiene methods should be individualized, and, therefore, each individual should be advised by his or her dentist or dental hygienist about which would be more suitable.

The results found in this review are generally consistent with the superiority of powered toothbrushes as compared to manual ones, especially concerning dental plaque removal and associated reduction of gingival inflammation.  Table 7 summarizes the evidence included in the present review in the different areas of interest assessed.

The studies included in this review observed the effects of powered toothbrushes on adults, children, individuals with special needs, and orthodontic patients. In general, the effect of powered toothbrushes was significantly superior to that of manual toothbrushes, with no evidence of a higher incidence of adverse effects.

The vast majority of included studies did not look for patient-centered outcomes, motivation, satisfaction, etc. In this sense, additional studies are warranted. The findings of this narrative review suggest that the powered toothbrush is a very promising alternative for self-performed plaque control.

## Figures and Tables

**Table 1:** Studies that compared the effectiveness of manual and powered toothbrushes on microbial biofilm.

Study (author, year)	Study design	Test group (toothbrush model, mechanism of action	Control group (toothbrush model, n male, n female, n smokers, mean age)	Brushing time	Outcomes of interest (clinical indexes)	Main results	Conclusion
Schmidt et al. [9]	3-species biofilm was formed in vitro on protein-coated titanium disks. Subsequently, biofilm-coated substrates were exposed to 4 different powered toothbrushes with side-to-side action for noncontact biofilm removal.	4 different powered toothbrushes with side-to-side action (A, B, C, D)	Brushing (bristle-to-disk)	2, 4, and 6 seconds	Biofilm volume as measured using a confocal laser scanning microscope.	Two powered toothbrushes with side-to-side action (C and D) promoted increased in vitro biofilm removal without bristle contact.	It is possible to reduce a three-species in vitro biofilm by noncontact brushing with two out of four side-to-side toothbrushes.
Schmidt et al. [10]	3-species biofilm was formed in vitro on protein-coated disks in an adjustable interdental space model. Subsequently, biofilm-coated substrates were exposed to three side-to-side powered toothbrushes with different oscillation frequencies for noncontact biofilm removal.	Oscillation frequencies of three commercial side-to-side toothbrushes were progressively reduced (100, 85, 70, 55, and 40%).	???	???	Biofilm volume as measured using a confocal laser scanning microscope.	The oscillation frequency of the tested side-to-side toothbrushes affected the biofilm reduction in an interdental space model.	Powered toothbrushes with side-to-side action set at higher oscillation frequencies may promote increased interdental biofilm removal.
Hope et al. [11]	Oral biofilms were grown in vitro on hydroxyapatite (HA) discs in an interdental space model. Next, biofilm-coated substrates were exposed to powered toothbrushes.	Sonicare Elite beyond the bristles and Braun Oral-B 3D	Nonoperating Sonicare Elite beyond the bristles and Braun Oral-B 3D	5 seconds	Biofilm volume as measured by reduction in bacterial counts.	Both brushes removed a significantly higher percentage of plaque bacteria compared to the inactive brushes (Braun, *p*=0.002; Sonicare, *p*=0.005). Percentage of plaque bacteria removed by the Sonicare Elite beyond the bristles (32.23%) was significantly greater (*p*=0.012) than that removed by the Braun Oral-B 3D (9.48%) in this model system.	Powered toothbrushes removed a significantly higher percentage of plaque bacteria compared to the inactive brushes

**Table 2:** Characteristics of studies that compared the effectiveness of manual and powered toothbrushes for gingivitis control.

Study (author, reference)	Study design	Test group (toothbrush model, mechanism of action, n male, n female, n smokers, mean age)	Control group (toothbrush model, n male, n female, n smokers, mean age)	Brushing time	Outcomes of interest (clinical indexes)	Experimental times	Main results
Grender et al. [12]	RCT, parallel, single-blind	Oral-B iO with Ultimate Clean brush head (oscillating/rotating) males: 18; females: 37; smokers: not reported; age: 46.1±12.8 (range:18-69)	Soft ADA manual toothbrush males: 15; females: 40; smokers: not reported; age: 48.3±15.8 (range: 19-83)	Twice daily	Rustogi Modification of the Navy Plaque Index (RMNPI), Modified Gingival Index (MGI), Gingival Bleeding Index (GBI), gingivitis status (“healthy”/“not healthy”)	Baseline, 1 and 8 weeks	At week 1, the powered brush group presented a significantly higher number of “healthy” subjects than the manual brush group (16.4% vs. 1.8%, *p*=0.008). This difference was pronounced at Week 8 (81.8% vs. 23.6%; *p*=0.001). Both brush groups had significant improvements from baseline at week 1 and week 8 for all three gingivitis measurements on the lingual surfaces, with *p*=0.011 on all measurements for the manual brush group and *p*=0.001 on all measurements for the powered brush group. For the between-group comparisons, there were significant differences seen at both week 1 (*p* ≤ 0.037 for all) and week 8 (*p* < 0.001 for all) favoring the powered brush group for all lingual gingivitis measures.
Bianco et al. [13]	RCT, parallel, single-blind. In patients diagnosed with lichen planus.	Philips FlexCare Platinum (sonic); males: 7; females: 9; smokers: 0; age: 61.0±9.3	GUM technique PRO soft 525; males: 5; females: 11; smokers: 0. Age: 65.4±11.1	3 min, twice daily	O'Leary Plaque Score, bleeding on probing (BOP). Angulated Bleeding Score (AngBS) and probing depth (PD)	Baseline 8 weeks	At 8 weeks, lower percentage of BOP-(*p* ≤ 0.001) positive sites and higher index of improvement in AngBS (*p* ≤ 0.014) and the extent and severity of desquamative gingivitis lesions in the sonic compared with the manual brush group (*p* ≤ 0.007). No significant differences were found between the two groups with regard to PD.
Mirza et al. [14]	RCT, parallel, single-blind.	Philips Sonicare DiamondClean Smart with Premium Plaque Control brush head (DCS) (sonic) n: 112; males: not reported; females: not reported; smokers: 0; age: not reported	Oral-B Genius 8000 with FlossAction brush head (OBG) n: 107; males: not reported; females: not reported; smokers: 0; age: not reported	2 min, twice daily	Lobene and Soparker modification of the Plaque Index Lobene Modification of the Gingival Index (MGI); van der Weijden modification of the Gingival Bleeding Index	Baseline 42 days	Gingivitis reduction and percent reduction per MGI following 42 days of product home use were 1.38 (1.30, 1.46) and 51.32% (48.45%, 54.19%) for DCS, and 0.53 (0.45, 0.61) and 20.07% (17.14%, 23.00%) for OBG. These results were statistically significant (*p* < 0.001).
Cahuana‐Vasquez et al. [15]	RCT, parallel, single-blind.	Oral‐B Vitality O‐R handle (D12) with an Oral‐B Sensi Ultrathin brush head (EB60) (oscillating/rotating); males: 29;females: 46; smokers: 6; age: 45.9±12.94	ADA manual toothbrush; males: 25; females: 50; smokers: 6; age: 45.5±12.93	2 min, twice daily	Rustogi modification of the Navy Plaque Index (RMNPI); Modified Gingival Index; Gingival Bleeding Index; number of bleeding sites	Baseline 5 weeks	MGI was reduced by 13.1% for the O‐R group and 5.4% for the manual group, and GBI was reduced by 54.0% and 23.3% for the O‐R and manual groups, respectively. Statistically, significantly greater reductions were observed for the O‐R brush versus the manual brush for MGI and GBI measures (*p* < 0.001). Mean bleeding site reductions from baseline at week 5 were 11.15 (52.2%) for the O‐R brush and 5.04 (23.6%) for the manual brush; the reduction was statistically significantly greater for the O‐R brush compared to the manual brush group (by 6.11, *p* < 0.001).
Garcia-Godoy et al. [16]	RCT, parallel, single-blind.	Oral-B Professional Care SmartSeries 5000 toothbrush with Oral-B CrossAction toothbrush head, D36/EB50 (oscillating/rotating) + two-step stannous fluoride dentifrice and hydrogen peroxide whitening gel system + expanded polytetrafluoroethylene floss; males: 9; females: 17; smokers: not reported; age: 36.6±11.80 (21-60)	Dental prophylaxis followed by use of standard sodium fluoride dentifrice and a manual Oral-B Indicator toothbrush; males: 6; females: 20; smokers: not reported; age: 34.9±10.80 (19-55)	2 min, twice daily	Turesky modification of the Quigley and Hein Plaque Index, marginal gingival bleeding	Baseline 2, 4, and 6 weeks	The number of bleeding sites in the experimental group continued to decrease throughout the trial, whereas after Week 2, the control group showed an increasing trend. compared to the control group, the test group had 55% fewer bleeding sites at Week 2, 85% fewer bleeding sites at Week 4, and 98% fewer bleeding sites at Week 6, which were all highly significant differences (*p* < 0.0001).
Schmalz et al. [17]	RCT, parallel, single-blind.	- Professional CareTM 7000 [oscillating-rotating, (OR)] Philips Sonicare TM [sonic-active (SA)] *OR* males: 8; females: 38; smokers: 6; former smokers: 6; age: 22.98±2.52; *SA* males: 10; females: 35; smokers: 6; former smokers: 4; age: 23.66±2.95	Elmex®INTERX; males: 9; females: 39; smokers: 8; former smokers: 3; age: 23.70± 3.13	2 min, twice daily	Turesky modification of the Quigley and Hein Plaque Index; Löe and Silness Gingival Index (GI); Papilla Bleeding Index	Baseline 2, 4, and 12 weeks	After 12 weeks, gingival inflammation and plaque indices were comparable between all subgroups (*p* > 0.05).
Nathoo et al. [18]	RCT, parallel, single-blind.	- Colgate ProClinical C200 powered toothbrush with Triple Clean Brush Head (TC).- Colgate ProClinical C200 powered toothbrush with Sensitive Brush Head (S). (both sonic) *Triple Clean*; males: 13; females: 27; smokers: not reported; *Sensitive* males: 18; females: 22; smokers: not reported; age: 40. 83 (19-67)	Oral B Indicator manual; males: 18; females: 22; smokers: not reported; age: 41.93 (18-62)	2 min, twice daily	Rustogi modification of the Navy Plaque Index (RMNPI); Löe and Silness Gingival Index (GI); Gingivitis Severity Index.	Baseline, 4 weeks	After four weeks, all three toothbrushes provided statistically significant (*p* < 0.05) reductions in gingivitis and gingivitis severity. In comparison to the manual toothbrush, Toothbrush triple clean and Toothbrush Sensitive provided a statistically significant (p < 0.05) reduction in gingivitis (10.0x and 9.33x, respectively) and gingivitis severity (5.67x and 7.0x, respectively). There were no differences between Toothbrush TC and Toothbrush S in respect to either gingivitis or gingivitis severity after four weeks of use.
Gallob et al. [19]	RCT, parallel, single-blind.	Colgate® ProClinical® A1500 Power Toothbrush (distinct multi-directional cleaning action); males: 16; females: 23; smokers: not reported; age: 45.5 (19-69)	Oral-B Indicator; males: 5; females: 35; smokers: not reported; age: 53 (21-69)	2 min	Rustogi modification of the Navy Plaque Index (RMNPI); Talbott modification of the Löe and Silness Gingival Index; Gingivitis Severity Index	Baseline 4 and 12 weeks	Relative to the baseline score, after 4 weeks, powered toothbrushes demonstrated a statistically significant (*p* < 0.05) reduction in gingivitis, whereas manual toothbrush demonstrated a statistically significant (*p* < 0.05) increase in gingivitis. The powered toothbrush group exhibited a statistically significant (p < 0.001) reduction in gingivitis severity of 333% relative to the manual toothbrush group. Relative to the baseline score, after 12 weeks. The powered toothbrush group demonstrated a statistically significant (*p* < 0.05) reduction in gingivitis, whereas manual toothbrush demonstrated a statistically significant (*p* < 0.05) increase in gingivitis. The powered toothbrush group exhibited a statistically significant (*p* < 0.001) reduction in gingivitis of 400% relative to the manual toothbrush group.

Nathoo et al. [20]	RCT, parallel, single-blind.	Colgate ProClinical A1500 powered toothbrush with Triple Clean Brush Head (TC), auto mode (sonic). Males: 15; females: 25; smokers: not reported; age: 44 (18-65)	Oral B Indicator manual flat-trim; males: 10; females: 26; smokers: not reported; age: 42 (20-66)	2 min, twice daily	Rustogi modification of the Navy Plaque Index (RMNPI); Löe and Silness Gingival Index (GI); Gingivitis Severity Index	Baseline 4 and 12 weeks	*Reduction of gingivitis after four weeks.* Powered toothbrush with Triple Clean Brush Head (TC) exhibited a statistically significantly (*p* < 0.05) greater reduction in Gingival Index scores of 0.09 as compared to an increase of 0.01 for the manual toothbrush. Gingivitis severity. Powered toothbrush TC exhibited a statistically significantly (*p* < 0.05) greater reduction in Gingivitis Severity Index scores of 0.03 as compared to 0.01 for the manual toothbrush. *Reduction of gingivitis after twelve weeks*. Toothbrush TC exhibited a statistically significantly (*p* < 0.05) greater reduction in Gingival Index scores of 0.42 as compared to 0.06 for the manual toothbrush. Gingivitis severity. Toothbrush TC exhibited a statistically significantly (*p* < 0.05) greater reduction in Gingivitis Severity scores of 0.14 as compared to 0.04 for the manual toothbrush.

Bogren et al. [21]	Multi-center RCT, parallel, single-blind.	ROA-powered toothbrush (oscillating/rotating) + triclosan/copolymer/fluoride-containing dentifrice; males: 28; females: 37; smokers: 19; age: 60 (36 to 82)	Multitufted soft manual toothbrush + standard fluoride- containing dentifrice; males: 25; females: 38; smokers: 19; age: 58 (34 to 79)	Twice daily	Plaque was scored positive if detected using a probe. Bleeding on probing (BOP), Probing depth (PD)	Baseline1, 2, and 3 years	No statistically significant difference in the proportion of bleeding sites was observed between the two groups at any of the examination intervals. There were no statistically significant differences with regard to PPD alterations between the test and control groups at the various examination intervals (min).
Bogren et al. [22]	Multi-center RCT, parallel, single-blind.	Oral B powered toothbrush (oscillating/rotating) + triclosan/copolymer/fluoride-containing dentifrice; males: 32 females: 48; smokers: 17; age: 38 (22 to 73)	Multitufted soft manual toothbrush + standard fluoride-containing dentifrice; males: 25; females: 55; smokers: 10; age: 38 (24 to 63)	2 min, twice daily	Plaque was scored positive if detected using a probe. Bleeding on probing (BOP), probing depth (PD)	Baseline1, 2, and 3 years	The individual change in BOP between baseline and 3 years revealed that 48 subjects (60%) in the test group and 42 subjects (52%) in the control group exhibited a decrease and that 27 subjects (34%) and 16 subjects (20%), respectively, exhibited an increase. No significant difference in the proportion of subjects according to BOP change was observed between the two groups. No significant differences were found between the two groups with regard to PD.
Gugerli et al. [23]	RCT, parallel, single-blind.	Oral B Pro Care 8000 (D18/EB17) (oscillating/rotating) (group P); males: 16; females: 19; smokers: 16; age: 48.71±1.94	ADA manual toothbrush (group M); males: 16; females: 19; smokers: 12; age: 49.03±1.86	twice daily	Silness and Löe Plaque Index (PI); Löe and Silness Gingival Index (GI); bleeding on probing (BOP); probing depth (PD)	Baseline, 7, 14, and 28 days	Group P (Powered Toothbrush) had a significantly lower mean number of sites with BOP and the remaining sites showed PI >1 at days 14 and 28. Differences in the mean GI, the number of sites with GI >1, mean recession, mean PD, and the number of pockets >4 mm were not significant. A closer inspection indicated that differences between groups P and M (Manual toothbrush) seemed to be particularly pronounced at the lingual aspects of the lower arch. In this dentition segment, subjects in group P had an average PI of 0.38 (±±0.26) at day 28, whereas the average PI in group M (manual toothbrush) was 0.69 (±0.31).

McCracken et al. [24]	RCT, parallel, single-blind.	Oscillating/rotating powered toothbrush (not otherwise specified); males: 9; females: 7; smokers: 4; age: 49 (32-67)	Manual toothbrush (not otherwise specified) (n male—n females—n smokers; mean age) males: 9; females: 7; smokers: 6; age: 49 (32-68)	2 min, twice daily	Turesky modification of the Quigley and Hein Plaque Index; Saxer and Muhlemann Papilla Bleeding Index; probing depth (PD)	Baseline 3, 6, 10 and 16 months.	There was an improvement in the oral health of all patients in both groups with respect to the levels of plaque, probing depth, and bleeding. The patterns of probing depth and plaque reduction were similar between manual and powered toothbrushes. In contrast, a significant difference in gingival bleeding was detected in favor of the manual brush after 16 months.
Zimmer et al. [25]	RCT, parallel, single-blind.	Ultra Sonex Ultima (sonic/ultrasonic) total: 31; males: not reported; females: not reported; smokers: not reported; age: not reported	Aronal compact manual total: 32; males: not reported; females: not reported; smokers: not reported; age: not reported	3 min, twice daily	Turesky modification of the Quigley and Hein Plaque Index; Approximal Plaque Index (API); Papillary Bleeding Index	Baseline, 4, and 8 weeks	At 8 weeks, the API showed no difference between the manual and the powered toothbrush. The PBI showed similar results. A statistically significant difference was found after 4 (*p* = 0.01) and 8 weeks (*p* = 0.001). The final median PBI was 0.63 for the manual brush, and 0.29 for the Ultra Sonex Ultima. During the study period, the Friedmann test showed a statistically significant improvement for the PI and the PBI for the manual toothbrush and the Ultra Sonex Ultima (*p*=0.001).
Dentino et al. [26]	RCT, parallel, single-blind.	Braun Oral B D9, Ultra Plaque Remover (oscillating/rotating); males: 28; females: 48; smokers: 11; age: 32.2 (18-61)	ADA accepted standard soft bristle manual; males: 25; females: 56; smokers: 18; age: 31.8 (18-59)	2 min, twice daily	Turesky modification of the Quigley and Hein Plaque Index; Lobene Gingival Index; bleeding on probing (BOP); gingival crevicular fluid (GCF)	Baseline, 3, and 6 months	All 3 independent measures followed the same pattern for both brushes, and the differences were not statistically significant between treatments.
Mantokoudis et al. [27]	RCT, crossover, single-blind	- Braun Oral-B Plak Control Ultra (oscillating)- Braun Oral- B Plak Control 3D (oscillating + long-axis motion) *Plak Control Ultra*; males: 16; females: 10; smokers: not reported; age: 25 (23-41); *Plak Control 3D* males: 16; females: 10; smokers: not reported; age: 25 (23-41)	Paro médium (ESRO AG, CH-8880 Thalwil); males: 16; females: 10; smokers: not reported; age: 25 (23-41)	2 min, twice daily	Turesky modification of the Quigley and Hein Plaque Index; bleeding on probing (BOP)	BaselineN2 weeks after each group allocation (crossover)	Differences between the mean BOP with different brushing techniques were not statistically significant.
Haffajee et al. [28]	RCT, parallel, single-blind	Braun Oral B 3D Plaque Remover (oscillating/rotating/pulsating); males: 13; females: 9; smoking: not reported; age: 49±2	Crest Complete; males: 15; females: 11; smoking: not reported; age: 48±2	Twice daily	Turesky modification of the Quigley and Hein Plaque Index; Löe and Silness Gingival Index (GI); bleeding on probing (BOP); probing depth (PD)	Baseline, 3, and 6 months	Mean pocket depth, mean plaque index, and % of sites exhibiting BOP showed significant reductions from baseline to 3 and 6 months in both groups. Mean attachment level and mean GI were significantly reduced in the powered brushing group only. There were no significant differences between groups at any time point for most of the measured parameters. However, a greater proportion of subjects in the powered brushing group showed a decrease in the % of sites with BOP at 6 months compared with subjects using a manual brush (82% vs 69% for powered and manual groups, respectively). Mean PD showed significantly greater reductions between baseline and 6 months in lingual and mandibular areas in the powered toothbrush group.
Aass and Gjermo [29]	RCT, cross-over, single-blind	- Philips HP555- Philips Jordan 2-action Plaque Remover HP510 (rotating/oscillating, controlled pressure); males: 13; females: 37; smokers: not reported; age:50% < 35 years old and 50% > 35 years old	Jordan V-shape, medium; males: 13; females: 37; smokers: not reported; age: 50% < 35 years old and 50% > 35 years old	2 min, twice daily	Turesky modification of the Quigley and Hein Plaque Index; Löe and Silness Gingival Index (GI)	Baseline and 3 weeks after each group allocation (cross-over)	All surfaces: HP555 higher GI than manual (*p*=0.02) posterior proximal lingual surfaces: HP555 higher GI than manual (*p*=0.02) and HP555 higher GI than HP510 (*p*=0.03)
Heasman et al. [30]	RCT, parallel, single-blind	- Braun Oral B D7- Philips/Jordan HP 735 (oscillating) *Braun Oral B D7* total: 25; males: not reported; females: not reported; smokers: not reported; age: not reported;*Philips Jordan HP 735* total: 25; males: not reported; females: not reported; smokers: not reported; age: not reported	Oral B Advantage B35 total: 24; males: not reported; females: not reported; smokers: not reported	At least 90 seconds, twice daily	Turesky modification of the Quigley and Hein Plaque Index; Löe and Silness Gingival Index (GI)	Baseline, 6 weeks	After 6 weeks, subjects using the Philips/Jordan HP 735 had lower full mouth (*p*=0.03), interproximal (*p*=0.08), and smooth surface inflammation scores (*p*=0.03) when compared to the other 2 groups. Within the group Philips/Jordan HP 735, however, the Gingival Index scores at 6 weeks are virtually identical to those at baseline. Within the other groups, all GI increased between baseline and the 6-week visit.
Ainamo et al. [31]	RCT, parallel, single-blind.	Braun Oral B Plak Control (oscillating/rotating); males: 32; females: 23; smokers: not reported; age: 39	Jordan manual toothbrush, soft; males: 32; females: 24; smokers: not reported; age: 37	2 min, twice daily	Ainamo and Bay Visible Plaque Index; Ainamo and Bay modified Gingival Bleeding Index	Baseline, 3, 6, and 12 months	At 6 and 12 months, the mean percentage of sites with bleeding on probing in the powered toothbrush was lower than in the manual (*p*=0.01). The differences tend to occur on the anterior labial and lingual surfaces. A drop in the mean percentage of sites with bleeding on probing at 6 and 12 months, when compared to the 3-month assessment was only detected in the powered toothbrush group (*p*=0.01).
van der Weijden [32]	RCT, parallel, single-blind.	Braun Plak Control (oscillating/rotating); males: 22; females: 20; smokers: not reported; age: 22.2	Butier GUM 311; males: 15; females: 20; smokers: not reported; age: 22.3	At least 2 min	Modification of the Quigley and Hein Plaque Index; Silness and Löe Plaque Index (PI); Vope Calculus Index; Lobene Modification of the Löe and Silness Gingival Index; marginal gingival bleeding	Baseline, 1, 2, 5, and 8 months	1 month later, during which the subjects had brushed according to written instructions, a decrease in all indices was observed, with the exception of bleeding in the manual group. No significant difference between groups was observed. At the 5-month appointment, a difference was observed for bleeding in favor of the powered toothbrush; although no difference was observed for visual signs of inflammation, groups show that the effect as observed at 5 months continued, and a significant difference between groups was observed for all but the calculus indices. The Braun Plak Control appeared to be more effective in the treatment of gingivitis.

Quirynen et al. [33]	RCT, cross-over, single-blind.	InterPlak toothbrush (rotating) gingivitis patients: males: 3; females: 3; smokers: not reported; age: 20-24; periodontitis patients: males: 4; females: 2; smokers: not reported; age: 30-59	Oral-B 30 toothbrush gingivitis patients: males: 3; females: 3; smokers: not reported; age: 20-24; periodontitis patients: males: 4; females: 2; smokers: not reported; age: 30-59	Twice daily	Quigley and Hein Plaque Index; Approximal Sites with Plaque (AP); Muhlemann and Son Sulcus Bleeding Index; probing depth (PD)	Baseline, 2, 4, 8, 13, 21, and 34 weeks	Gingivitis study: After 1 and 4 months the periodontal situation improved, with significantly more reduction in gingivitis and probing pocket depth for the regions cleaned with the powered toothbrush. When lingual sites or approximal surfaces were considered, even more significant differences supporting the use of the powered toothbrush appeared. Periodontitis study: the reduction of gingivitis was more significant with the Interplak toothbrush. After 34 weeks, gingival inflammation almost disappeared in these quadrants. Brushing with the Interplak toothbrush also resulted in a greater pocket reduction when compared to the manual brushing.

**Table 3:** Summary of studies that have addressed adverse effects of powered versus manual toothbrushes.

Study (author, reference)	Study design	Test group (toothbrush model, mechanism of action, *n* male, *n* female, *n* smokers, mean age)	Control group (toothbrush model, *n* male, *n* female, *n* smokers, mean age)	Brushing time	Outcomes of interest (clinical indexes)	Experimental times	Main results
Salzer et al. [38]	Randomized controlled clinical trial in individuals susceptible to gingival recession	Different types of toothbrush	n=55, manual toothbrush	Twice daily for 2 min	Influence on pre-existing GR following 12 months of toothbrushing with a power toothbrush compared to a reference manual toothbrush	12 months	Neither the power toothbrush nor manual toothbrush led to an increase in pre-existing gingival recession during 12 months of daily use.
Dorfer et al. [39]	Randomized controlled clinical trial in subjects with pre-existing gingival recession	Different types of toothbrush	n=54, ADA reference manual toothbrush	2 min	Long-term effects of brushing with an oscillating-rotating powered toothbrush vs. an ADA reference manual toothbrush on pre-existing gingival recession	3 years	Gingival recession in subjects with pre-existing recession was significantly reduced after 3 years of brushing with either a powered or manual toothbrush.
McCracken et al. [40]	Randomized controlled clinical trial in healthy patients with pre-existing gingival recession of at least 1mm	Different types of toothbrush	n=26, manual toothbrush	Twice daily for 2 min	Clinical effects of manual vs. powered toothbrushes on sites of localized gingival recession	12 months	No progression of gingival recession in subjects using either toothbrush over 12 months. No difference in the overall wear of the powered and manual toothbrushes over successive 3-month periods.
Hefti and Stone [36]	Randomized controlled clinical trial in patients complaining from tooth hypersensitivity in canines or premolars	Different powered toothbrushes		Twice daily for exactly 2 min	Effect of different types of powered brushes in a clinical trial designed for the study of dentin hypersensitivity	8 weeks	Treatment-related differences were not statistically significant.
Rosema et al. [41]	Randomized controlled clinical trial	Different powered toothbrushes	n=90, manual brush		To assess gingival recession (GR) in manual and powered toothbrush users and evaluate the relationship between GR and gingival abrasion scores	At least 1 year	There was no correlation between gingival abrasion as a result of brushing and the observed gingival recession following use of either toothbrush.

**Table 4:** Characteristics of studies that compared the effectiveness of manual and powered toothbrushes in youngsters.

Study (author, reference)	Study design	Test group (toothbrush model, mechanism of action, n subjects)	Control group (toothbrush model)	Brush time	Outcome of interest (clinical indexes)	Experimental times	Main results
Erbe et al. [42]	RCT, replicate single-use, two-treatment, four-period, crossover, examiner-blind study design	Oscillating-rotating powered toothbrush with orthodontic brush head (Oral-B Triumph, D27/OD17, Procter & Gamble, Cincinnati, Ohio) and sonic toothbrush (Sonicare FlexCare with ProResults brush head, HX6011, Philips Oral Healthcare Inc, Bothell,)N = 44; 17; females 27 males; age: 12 and 25 years	Oscillating-rotating powered toothbrush with orthodontic brush head and sonic toothbrush	2 minutes for each brushing, alternating the brushes morning and evening.	Plaque was measured, using DPIA The data were analysed using SAS software (SAS Institute Inc., Cary, NC, USA	1 week	Baseline plaque levels for both brush treatments were high, covering more than 50% of the tooth area. Effective plaque removal was observed with both toothbrushes (*p* < 0.001); however, the reduction in plaque with the oscillating-rotating toothbrush was statistically significantly greater (*p* < 0.017) compared with the sonic toothbrush.
Mascarenhas et al. [43]		Battery-powered toothbrush (Oral-B Kids) N = 30; all males; age: 9 to 11 years	Manual toothbrush (not otherwise specified)	Not reported	Plaque buildup was assessed using the Soparkar modification of the Quigley and Hein Plaque Index.	Two weeks	At baseline, there was no difference in plaque removal between battery-powered tooth brushing and manual tooth brushing, either in difference between pre- and post-tooth brushing plaque measures (*p*=0.44) or in percentage change (*p*=0.51). After two weeks of use, there was a statistically significant difference in plaque removal between battery-powered tooth brushing and manual tooth brushing, both in the difference between pre- and post-tooth brushing plaque measures (*p*=0.01) and in percentage change (*p*=0.006). Mean plaque removal by manual tooth brushing was 0.97 ± 0.45, and mean plaque removal by battery-powered toothbrush was 1.23 +± 0.56. Mean percentage change in plaque removal by manual tooth brushing was 33.5 ± 16.05, and mean plaque removal by battery-powered tooth brushing was 43.0 ± 18.82, which represented a 9.5% improvement for battery-powered tooth brushing compared to manual tooth brushing.

Kallar et al. [44]	Comparative clinical study	Powered toothbrush (not otherwise specified); N=200; age: 6 to 13 years	Manual toothbrush (not otherwise specified)	Not reported	Plaque was recorded according to the Turesky-Gilmore-Glickman modification of Quigley and Hein Index and oral hygiene performance index	3, 6, 9, and 12 weeks	Powered brushes showed significant plaque reduction as compared to the manual brushes. Supervised group of both brushes showed a greater plaque reduction.
Ghassemi et al. [45]	Randomized crossover clinical trial	Spinbrush GLOBRUSH, (Arm&Hammer Co, Inc. Princeton, NJ, USA); N = 105; 8-12 years (52 subjects); 13-17 years (53 subjects)	Oral-B Indicator 30 compact soft toothbrush (Procter & Gamble Co., Cincinnati, OH, USA)	Two minutes	MNPI SCORES to evaluate the difference between the subjects' pre- and post-brushing mean ANCOVA	One week	Between-group analyses showed that the powered brush produced a statistically significantly greater plaque reduction than the manual brush, both whole mouth (12.8%, *p* < 0.0001) and at all subset sites, including difficult-to-reach areas such as the posterior lingual gingival region (74.9% greater plaque reduction, *p* < 0.0001).
Davidovich et al. [46]	Randomized clinical study	Oral-B Pro-Health For Me Vitality power toothbrush (D12 kids' handle and EB17 soft brush head; Procter & Gamble, Cincinnati, OH, USA); N=41; age 8-11 years	Oral-B Pro-Expert Cross Action 8+ (OK 011) soft manual toothbrush (Procter & Gamble, Cincinnati, OH, USA)	Two minutes	To quantify pre-brushing existing plaque, two clinical examiners conducted a whole-mouth Turesky-modiﬁed Quigley and Hein Plaque Index (TMQHPI) examination.	One week	Both the powered toothbrush and manual toothbrush provided statistically signiﬁcant mean plaque reductions as compared to baseline in all analyses (p < 0.001). For both examiners, plaque removal was signiﬁcantly (p < 0.001) greater for the power toothbrush in permanent and mixed dentitions. The inter-examiner correlations for the permanent dentition were strong (ICC = 0.68-0.88) for pre-brushing plaque across all periods.

**Table 5:** Characteristics of studies that compared the effectiveness of powered toothbrushes in individuals with special needs.

Study (author, reference)	Study design	Test group (toothbrush model, mechanism of action, n subject)	Control group (toothbrush model, n subject )	Inclusion criteria	Brushing time	Outcomes of interest (clinical indexes)	Experimental times	Main result
Vajawat et al. [47]	Clinical microbiological study	Colgate 360° sonic power toothbrush, Colgate-Palmolive Company, New York, NY, USA (sonic);N = 20; age: mean, 18.6 years	Colgate 360° toothbrush, Colgate-Palmolive Company, New York, NY, USA) N = 20; age: mean, 17.7 years	Patients diagnosed with autism in the age group ≥15 years, with a minimum of 20 teeth, no prior experience of using a powered toothbrush, and willing to participate were selected.	3 minutes	ANOVA was used to compare the mean plaque and gingival scores between the test and control groups. Chi-square test and McNemar test were used to assess the difference between the detection rates of P. gingivalis, T. forsythia, and T. denticola at baseline and 4 weeks.	at baseline, 1, 4, and 12 weeks	In patients with autism spectrum disorder, powered toothbrushes result in a significant overall improvement in plaque control and gingival health, when constant reinforcement of OHI is given. However, there was no difference in the detection rate of red complex organisms between the groups.
Silva et al. [49]	Randomized crossover trial	Techline EDA-01, Techline, São Paulo, Brazil (powered oscillating/rotating); N = 16; age: 6 to 14 years. ^*∗*^Each participant used one of the toothbrushes and then, after a 7-day washout period, used the other type.	Dental Brush Medfio Slide Pro, Medfio, Pinhais, Brazil; N = 16; age: 6 to 14 years	Having good periodontal health confirmed by the dentist responsible for the individuals' oral health care, being between six and 14 years old, 25 and having at least one tooth in each sextant.	2 minutes	The Quigley Hein Index (modified by Turesky et al.) was used to quantify biofilm. Basic fuchsin dye solution (Replak ® , Dentsply, York, USA) was used before and after each toothbrushing. Behavioral assessment was carried out using the Frankl Behavior Scale	7-day period with each type with 7-day washout period in between.	The use of powered or manual toothbrush had no effect on the quantity of dental biofilm removed in children and teenagers with DS, nor did it influence their cooperation during the procedure.
García-Carrillo et al. [50]	Cluster-randomized clinical trial	Sonicare EasyClean, Philips, Eindhoven, The Netherlands (sonic); 17 males; 11 females; 4 smokers; age: mean, 34.5 years	Vitis Access, Dentaid, Barcelona, Spain; 17 males; 7 females; 8 smokers; age: mean, 34.5 years	Adults with intellectual disability categorized as borderline [intelligence quotient (IQ) >70], mild (IQ, 50-69), or moderate (IQ 35-49). Being part of psychosocial support groups under the supervision of a trained monitor (special educators, with different university degrees).	2 minutes	For cluster-level analyses, demographical data were analyzed by ANOVA for continuous variables and chi-square test for categorical data. Clinical outcome variables were analyzed by repeated measures ANCOVA considering time and group as factors and respective baseline values as covariates (generalized linear model).	6-month trial	The tested sonic powered toothbrush was as effective and safe as the manual toothbrush. The use of powered or manual toothbrushes, together with fluoride toothpaste, may improve plaque and gingivitis levels, in patients with mild to limited intellectual disability.
Doğan et al. [51]	Comparative study	New manual toothbrush (CrossAction; Oral‐B [35 compact, 40 regular]) and powered toothbrush with an oscillating rotating head (Braun Plaque Control 3D [Braun 3D]; Oral‐B [D15525]); N = 15; age: 6-12 years	Manual triple‐headed brush (SuperBrush; Dento Co. AS [junior, regular]) N = 15; age: 13-18 years	Mentally challenged individuals with no history of receiving antibiotic and/or antiseptic therapy. No use of supplemental plaque control aids over the previous 5 months A minimum of 20 teeth present with no interposed edentulous spaces or loss of interdental contacts. In same range of intelligence and ability to brush their own teeth	3 minutes	The Quigley and Hein (QH) Plaque Index and the Approximal Plaque Index (API) were used to assess the oral hygiene status of each participant.	After 1 week of application, a week of washout before each group switched to the next type of toothbrush. The study lasted for 5 weeks.	The study indicated that the powered toothbrush is the most effective for removing dental plaque in mentally disabled children, whereas the SuperBrush is a good alternative
Vandana et al. [52]	Comparative evaluation, subject as own control	Colgate 360° sonic brush; 20 males; 10 females; age: 15 and 30 years	Manual brushing (MB) in its inactive state (power button: off). (Colgate 360° sonic brush) 20 males; 10 females; age: 15 and 30 years	Patients with at least 20 teeth, pre-brushing score of 1.5 or more (modification of Quigley-Hein Plaque Index [QPI]), mild to moderate gingivitis (according to modified gingival index [MGI], scoring criteria: 2, 3) and mild to moderate degree of mental retardation (according to Stanford-Binet scale: mild retardation: 55-69, moderate retardation: 40-54).	2-3 minutes	The clinical and microbial parameters recorded were subjected to statistical analysis using Mann-Whitney and Pearson correlation tests.	The recording of all clinical and microbial parameters was done on day 0 and day 21, while the clinical parameters were recorded up to day 45.	On intragroup comparison, throughout the study phases, both manual and powered brushing significantly reduced the Quigley-Hein Plaque Index (48%), Gingival Bleeding Index (GBI) (44%), and modified Gingival Index (52%). The Pearson correlation between GBI and periodontal pathogens like Prevotella internedia, Porphyromonas gingivalis, and Fusobacterium nucleatum showed statistically significant relation (p < 0.05) in the powered brushing group.
Goyal et al. [48]	Randomized crossover clinical trial	Oral-B Cross Action Power (7200 rpm: medium) N = 16; 10 males; 6 females; age: 15-25 years. ^*∗*^ In group A, manual toothbrush was assigned for the first 3 months followed by powered toothbrush for next 3 months. The order was reversed in group B—powered toothbrush was used for the first 3 months, followed by manual toothbrush for the next 3 months	Manual toothbrush N=16; 10 males; 6 females; age: 15-25 years	Mentally challenged patients with at least 20 teeth. Patients whose parents/ principal will sign on consent form.	2-3 minutes.	The clinical scoring procedure was used to assess plaque formation was the plaque index (Sillness and Loe, 1964) and to assess gingivitis was the Loe and Sillness Gingival Index (L-S index, 1963).	6-month evaluation of the plaque and gingival scores was done at the end of 1, 2, and 3 months for both the groups.	For mentally challenged individuals, manual toothbrushes reinforced with audiovisual instructions for brushing may be comparable to the use of powered toothbrushes. Comparison of mean plaque and gingival scores of manual and powered toothbrushes at different intervals in both groups were not statistically significant.
Smith et al. [53]	Randomized clinical trial	Sonicare Advance 4300, Philips Oral Healthcare, Snoqualmie, Wash., USA (sonic) at least twice a day; N = 11; age: under 10 years	Usual home care with manual brushes at least twice a day; N = 12; age: under 10 years	Patients that had received a renal transplant more than 1 year previously		Descriptive analyses were used to report group characteristics, and group comparability was examined by t-test (age) and chi-square (gender, race/ethnicity, time since transplantation, use of calcium channel blockers, baseline oral hygiene, and baseline DIGO), with significant differences noted at *p*=0.05.	12 months	After 12 months, the control group had significantly more severe pediatric patients with drug-induced gingival overgrowth than did the sonic tooth brushing and oral hygiene instruction group. Of the risk factors considered, only male gender was significantly associated with worse outcome. The use of a powered toothbrush, together with oral hygiene instruction, may be an important component of health maintenance for pediatric transplant patients on ciclosporin.

**Table 6:** Characteristics of studies that compared the effectiveness of powered toothbrushes in individuals undergoing orthodontic therapy.

Study (author, reference)	Study design	Test group (toothbrush model, mechanism of action, n male, n female, n smokers, mean age)	Control group (toothbrush model, n male, n female, n smokers, mean age)	Inclusion criteria	Brushing time	Outcomes of interest (clinical indexes)	Experimental times	Main results
Erbe et al. [56]	Parallel-group, randomized, active- controlled trial with a 1:1 allocation ratio.	Oral-B Professional Care 6000, D36/EB20 (oscillating/ rotating) males: 15; females: 15; no smokers; age: 13-17 years	Oral-B Indicator 35 soft; males: 15; females: 15; no smokers; age: 13-17 years	Good health and undergoing full upper and lower arch orthodontic appliance therapy	Twice daily for at least 2 minutes	Turesky-modified Quigley and Hein Plaque Index (TMQHPI) Modified Quigley and Hein Index (MQH)	Baseline, 2, and 6 weeks	Fifty-nine subjects aged 13-17 years completed the study. The interactive powered toothbrush provided significantly (p < 0.001) greater plaque reduction versus the manual toothbrush at 2 and 6 weeks according to the whole-mouth TMQHPI. The treatment difference in adjusted mean plaque change from baseline was 0.777 (95% CI, 0.614-0.940) at week 2 and 0.834 (0.686-0.981) at week 6. Mean reductions in the number of focus care areas were also significantly greater (p < 0.001) in the power brush group at weeks 2 and 6. Brushing times increased significantly at weeks 2 and 6 (p ≤ 0.013) versus baseline in the interactive power brush group only. Subject-reported motivation was significantly increased in the interactive power brush group at week 6 versus screening (p ≤ 0.005).
Erbe et al. [57]	Replicate-use, single-brushing, 3-treatment, examiner-blind, randomized trial	Oscillating-rotating powered toothbrush with a specially designed orthodontic brush head (Oral-B Triumph, OD17; Procter & Gamble, Cincinnati, Ohio); males: 29 females: 17; smokers: not reported; age: 12-25 years (mean: 14.6)	The same powered toothbrush handle with a regular brush head (EB25; Procter & Gamble); and a regular ADA manual toothbrush	Good health and undergoing full upper and lower arch orthodontic appliance therapy	6-period crossover study with washout periods of approximately 24 hours between visits	Turesky modified Quigley and Hein Plaque Index (TMQHPI)	Baseline w/DPIA, and postbrushing w/DPIA	The powered toothbrush, with either brush head, demonstrated significantly greater plaque removal over the manual brush. The orthodontic brush head was superior to the regular head
Costa et al. [58]	Single-blind crossover study	Ultrasonex Ultima Toothbrush (Sonex International, Brewster, NY);N = 21(11 boys, 10 girls; age range, 12-18 years; mean, 15.2 6 1.7 years)No smokers	Powered brush (Braun Oral B 3D Plaque Remover, Braun GmbH, Kronberg, Germany)Manual brush (Oral B Model 30, Gillete do Brasil, Manaus, Brazil)The participants were randomly divided into 3 sequences of brush use: (1) ultrasonic, powered, and manual; (2) manual, ultrasonic, and powered; and (3) powered, manual, and ultrasonic.	Good health and undergoing full upper and lower arch orthodontic appliance therapy	The subjects were asked to use their assigned toothbrush 3 times daily for 2 minutes with a designated toothpaste	Silness-Löe Plaque Index	For each crossover, patients used a toothbrush for 30 days, followed by a washout period of 14 days	Although counts and prevalences of some taxa examined decreased in the 3 groups, no toothbrush demonstrated superiority, when used three times daily for 2 minutes, on microbiologic parameters in banded molars of adolescent orthodontic patients. Furthermore, more comprehensive studies with other experimental designs are needed to determine whether these results can be sustained.
Thienpont et al. [59]	Prospective single-blind crossover clinical trial	Braun Oral-B 3D Plaque Remover (Kronberg, Germany) Philips-Jordan HP 510 (Philips Domestic Appliances, Groningen, The Netherlands); males: 18; females: 18; reported age: 11-24 years (mean: 13.6 years)	Lactona orthodontic toothbrush (Bergen op Zoom, The Netherlands) Oral-B Advantage Control Grip (Braun)	Good health and undergoing full upper and lower arch orthodontic appliance therapy	All 33 patients used each of the 4 brush types for 1 month in a randomly designed sequence over the course of a year.	Modified Gingival Index (GI); Bleeding on Probing Index (BOPI); Plaque Index, tooth (PIT); and brackets (PIB)	Baseline and after every 4-week test period	No statistically significant differences were found for any of the parameters measured after analysing scores for the upper and lower jaws. This indicates that no brush was less efficient than the others for either the upper or the lower jaw. For the left and right sides, the 4 brushes were also found equally efficient for all parameters, except for the PIT on the left side. For this index, the Philips-Jordan toothbrush had the best score.
Heasman et al. [60]	Three period, single-blind, cross-over trial	Dental Logic HP550 with regular brush head HP5924 (Philips, U.K.) Braun Oral B Plaque Remover (D7) with dedicated orthodontic brush head OD5-1 (Braun AG, Germany); N = 6021 males; 39 females; age 10-16 years (mean: 13.6, SD 1.2 years)	Manual dedicated orthodontic toothbrush (P35, Oral B Laboratories, Calif.)	Good health and undergoing full upper and lower arch orthodontic appliance therapy	The first brush was given 2 weeks after baseline (visit 1). The time interval for using each brush was 4 weeks at the end of which visible plaque and gingival bleeding indexes were recorded and a further prophylaxis given	Visible Plaque Index (VPI); GBI, Gingival Bleeding Index	The time interval for using each brush was 4 weeks from baseline	This 4-week, cross-over study demonstrated that the Philips HP550, the dedicated powered Braun D7, and the manual (Oral B) orthodontic brushes were equally effective in removing plaque and reducing gingival inflammation as indicated by bleeding on probing in patients undergoing fixed orthodontic treatment.
Trimpeneers et al. [55]	Single-blind, cross-over, clinical trial	Interplak (Bausch& Lomb) Philips (Philips) 36 orthodontic subjects; age: 11 years and 5 months to 15 years and 2 months (mean: 12 years and 10 months); 19 girls (mean age:12 years and 7 months); 17 boys (mean age: 13 years and 1 month)	Rotadent (Novitas), powered Blend-a-Med, (Procter & Gamble), manual	36 systemically healthy patients with orthodontic fixed appliances	3 minutes	Gingival Index; Bleeding Index; Plaque Index (PI); Plaque Index brackets (PIb).	At baseline and after 1 and 2 months, all clinical parameters were measured.	Manual toothbrushes presented significantly better effect than powered toothbrushes. Of the three powered toothbrushes tested, the Philips toothbrush seemed to give slightly better results than the Interplak toothbrush, whereas Rota-dent very clearly gave results inferior to all others.
Heintze et al. [61]	Single-blind “Latin square design” study	Interplak (Bausch & Lomb, Berlin, Germany), Rota-dent (Rota-dent, Küsnacht, Switzerland), Braun Oral-B Plaque Remover (Braun/Oral-B, Kronberg, Germany). N=38; median age 15.3 years	Elmex 29, Wybert GmbH, Lörrach, Germany A manual technique	Healthy subjects using fixed orthodontic appliances	Twice per day, 2-minute brushing	O'Leary Plaque Index (PI) and Ainamo Gingival Bleeding Index (GBI),	Patients were randomly allocated to groups who, within the test period, alternately used the toothbrushes. Before getting a new toothbrush that was to be used for a period of 4 weeks, each patient received video and written instructions. For another 4 weeks, the patient returned to the usual oral hygiene procedures before receiving the next new toothbrush.	Under home conditions, Rota-dent, without additional devices, can contribute to the improvement of oral hygiene in orthodontic patients compared with a manual technique with a hand toothbrush, interdental brush, and dental floss. The same holds true for the Braun Oral-B Plaque Remover with the orthodontic head, but only for patients with poor oral hygiene. Powered toothbrushes can improve patient motivation, which is very valuable over a treatment time of 2 years or more. Orthodontists are well advised to not rely solely on new powered devices but rather focus on enhancing the patient's dental awareness of oral hygiene.
Boyd and Rose [54]	Single-blind evaluation	Rota-dent, Prodentec Corp., Batesville, Ark.) 30 subjects; ninety consecutive adolescent patients who were to receive orthodontic treatment. There were 22 female and 13 male patients in the control group, 19 female and 11 male patients in the first treatment group, and 15 female and 10 male patients in second treatment group.	Manual toothbrushing only (control group); 25 subjects; once-daily use of a 0.05% NaF, 0.05% sodium fluoride rinse 20 subjects	Healthy subjects using fixed orthodontic appliances	Twice per day, 2-minute brushing	Plaque lndex; Gingival Index; Bleeding Index	All subjects in both treatment groups, but not the control group, were also instructed to use a 0.05% NaF mint-flavored mouth rinse (Flurigard, Colgate) once a day at bedtime. They were told to keep half ounce of the rinse in their mouth for 1 minute and then to expectorate, but not rinse with water, after using the rinse. These instructions also were reinforced at each monthly visit.	The results showed that although there were no significant differences between the three groups at baseline, the Rota-dent group showed significantly (*p* < 0.05) less post-treatment decalcification than either the control or rinse groups. In a separate analysis of first molars, the Rota-dent group again showed the least decalcification and the control group showed the most.

**Table 7:** Summary of the evidence supporting the comparison of powered versus manual toothbrushes.

Topic of interest	N (%) of included studies that support superiority of powered vs. manual toothbrushes	N (%) of included studies that support equivalence of powered and manual toothbrushes	N (%) of included studies that support superiority of manual vs. powered toothbrushes
Effects on dental plaque/gingival inflammation in adults	15 (68.2%)	5 (22.7%)	2 (9.1%)
Adverse effects in adults	0 (0%)	5 (100%)	0 (0%)
Effects on dental plaque/gingival inflammation in children	2 (40%)	3 (60%)	0 (0%)
Effects on dental plaque/gingival inflammation in special-needs individuals	3 (42.9%)	4 (57.1%)	0 (0%)
Effects on dental plaque/gingival inflammation in patients under fixed orthodontic therapy	4 (50%)	3 (37.5%)	1 (12.5%)

## Data Availability

The data are available on Pubmed.
